# Comparison of physical activity questionnaires for the elderly with the International Classification of Functioning, Disability and Health (ICF) – an analysis of content

**DOI:** 10.1186/s12889-015-1562-3

**Published:** 2015-03-14

**Authors:** Katharina G Eckert, Martin A Lange

**Affiliations:** Institute of Exercise and Public Health, Faculty of Sport Science, University Leipzig, Jahnallee 59, 04109 Leipzig, Germany

**Keywords:** Physical activity, Elderly, Questionnaires, Content analysis, International Classification of Functioning, Disability and Health (ICF)

## Abstract

**Background:**

Physical activity questionnaires (PAQ) have been extensively used to determine physical activity (PA) levels. Most PAQ are derived from an energy expenditure-based perspective and assess activities with a certain intensity level. Activities with a moderate or vigorous intensity level are predominantly used to determine a person’s PA level in terms of quantity. Studies show that the time spent engaging in moderate and vigorous intensity PA does not appropriately reflect the actual PA behavior of older people because they perform more functional, everyday activities. Those functional activities are more likely to be considered low-intense and represent an important qualitative health-promoting activity. For the elderly, functional, light intensity activities are of special interest but are assessed differently in terms of quantity and quality. The aim was to analyze the content of PAQ for the elderly.

**Methods:**

N = 18 sufficiently validated PAQ applicable to adults (60+) were included. Each item (N = 414) was linked to the corresponding code of the International Classification of Functioning, Disability and Health (ICF) using established linking rules. Kappa statistics were calculated to determine rater agreement.

**Results:**

Items were linked to 598 ICF codes and 62 different ICF categories. A total of 43.72% of the codes were for sports-related activities and 14.25% for walking-related activities. Only 9.18% of all codes were related to household tasks. Light intensity, functional activities are emphasized differently and are underrepresented in most cases. Additionally, sedentary activities are underrepresented (5.55%). κ coefficients were acceptable for n = 16 questionnaires (0.48-1.00).

**Conclusions:**

There is a large inconsistency in the understandings of PA in elderly. Further research should focus (1) on a conceptual understanding of PA in terms of the behavior of the elderly and (2) on developing questionnaires that inquire functional, light intensity PA, as well as sedentary activities more explicitly.

## Background

The evidence regarding correlations between physical activity and health-related outcomes for older people has rapidly grown in the last decade. Physical activity can help maintain overall health, recover from injuries faster, and slow down age-related decline in physical and mental functioning [1,2] or the progress of chronic diseases [3,4]. In the context of advanced age, functional activities in everyday life such as carrying groceries, climbing stairs or going for a walk become more relevant than other activities, e.g., sports activities, because those activities maintain an individual’s independence and health-related quality of life [5,6].

The growing scientific and applicatory interest in the field of physical activity has led to an extensive endeavor resulting in a variety of physical activity questionnaires (PAQ) that assess physical activity. The main purpose of these questionnaires is to assess the amount of time spent on and frequency of a certain activity in the last 5 up to 365 days. Often information of the intensity level of the activities is also retrieved through questionnaires [7]. However, there are questions regarding several aspects of their validity.

Most existing questionnaires are derived from an energy expenditure-based perspective and assess activities with a certain intensity level because evidence connects higher levels of energy expenditure with a variety of health-promoting effects [8]. The consequence of this emphasis is that activities with a moderate or vigorous intensity level, e.g., sports activities, are predominantly used to determine a person’s physical activity level in terms of quantity [5]. Studies show that the time spent engaging in moderate and vigorous intensity physical activity does not appropriately reflect the actual physical activity behavior of older people because they perform more functional, everyday activities [6,9]. Those functional activities are more likely to be considered low-intensity physical activity and represent an important qualitative health-promoting activity [10,11]. Therefore, focusing mainly on activities with a higher rate of energy expenditure leads to a very specific and narrow view of physical activity that misses the majority of activities in which elderly people take part and can generate floor effects by excluding less intense activities [12,13]. In addition, sedentary behavior has emerged as an independent risk factor for health among older adults [14], but is not sufficiently covered by most of the questionnaires.

A second aspect of consideration that does not only occur in PAQ for the elderly but is more noticeable in these measurement instruments is the highly variable content of PAQ [15]. In the context of physical activity, Pettee Gabriel et al. [16] presented a conceptual framework defining physical activity as “a complex and multidimensional behavior that does not stand in isolation from other related constructs, including sedentary behavior, energy expenditure, and physical fitness” (p.15). Based on their proposed conceptual framework, the authors specifically understand physical activity “as the behavior that involves human movement, resulting in physiological attributes including increased energy expenditure and improved physical fitness”. Within that framework, four general domains have been noted, (a) leisure time physical activities, (b) work- or school-related activities, (C) household, and (d) transport activities. The global construct of interest in this model is “human movement”. It reflects the directional relationship between the behavioral aspect of human movement, the characteristic, and the physiological result (energy) of movement for the first time [16].

However, although the authors present a definition and conceptual basis of the four domains mentioned above, the term ‘physical activity’ remains broad and wide ranging, especially in regard to generating and interpreting the outcome. Williams et al. [17] identified 104 questionnaires that measure physical activity in elderly and chronically ill populations and analyzed the content and format of those instruments on the domain level. The analysis notes a broad lack of agreement regarding content and format. To date, there is no consistency about exactly how to measure physical activity in the elderly or, in other words, “what to ask”. A more detailed analysis of questionnaires not only on a domain level but on an item level has to be done.

The aim of the study was to analyze the content of PAQ for the elderly. Specifically, we want to (1) identify common PAQ for the elderly, (2) link every item to the International Classification of Functioning, Disability and Health (ICF), (3) analyze their content and finally (4) discuss the findings with regard to the described conceptual framework. The findings should support users in choosing an adequate instrument for a specific context or purpose.

## Methods

### Selection of measures

**Figure 1:**
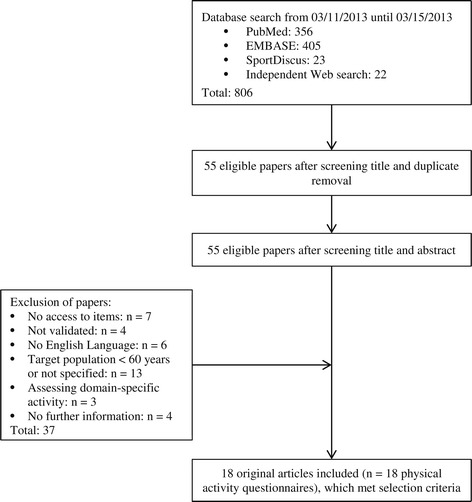
**Flow diagram of the search procedure.**

The research approach was based on the PRISMA guidelines (Preferred Reporting Items for Systematic Reviews and Meta-Analyses) [18]. The literature search took place in March 2013 using three major databases for social sciences (*PubMed*, *EBSCO HOST* and *SportDiscus*). We searched for PAQ using the following keywords with Boolean operators (AND, OR) in titles and abstracts: “questionnaire”, “scale”, “index”, “physical activity”, “activity”, “sport”, “exercise”, “adults”, “adolescents”, “older” and “elderly”. 784 articles were identified through the database search and an additional 22 articles through independent web research (N = 806). We removed 751 duplicates, which were mainly validation studies in different populations, settings or validation approaches. The remaining 55 articles were screened for English language and full access to the questionnaire’s items (see Figure 1). Also the applicability for older adults had to be given by either explicitly mentioning the target group of elderly or being validated within the age range of 60 to 90 years. Three PAQ were excluded, the *Occupational Physical Activity Questionnaire* (OPAC) [19], the *Occupational Sitting and Physical Activity Questionnaire* (OSPAQ) [20] and the *Tecumseh Occupational Physical Activity Questionnaire* (Tecumseh OPAQ) [21] because they were only applicable in occupational contexts. Due to the fact that we wanted to analyze the content of questionnaires assessing the physical activity levels of older people, the administration mode was less relevant. Therefore, we included both interview-based and self-administered instruments.

The remaining PAQ (n = 18) contained a total of 414 items (every item refers to a question) and 85 context based physical activity categories varying from 8 to 77 items and 1 to 10 domains per instrument. The characteristics of the included PAQ are presented in Table 1.

**Table 1:** **Characteristics of included physical activity questionnaires for the elderly**

**Physical Activity Questionnaire**	**Abbreviation**	**Total items**	**Administration mode**	**Studies on validity**	**Year**	**Target group (in years)**	**Recall period (in days)**
7-Day Physical Activity Recall [22]	7-Day PAR	10	IB	Cr.V	1985	20 to 74	7
Australian Activity Survey [23]	AAS	13	SA	Cr.V, Cs.V	2003	18 to 75	7
Baecke modified physical activity questionnaire for the elderly [24]	Baecke modified	12	SA	Cr.V	1991	63 to 80	7
Behavioral Risk Factor Surveillance System [25]	BRFSS	8	IB	Cr.V	2012	18 and older	7 or 31
Brunel lifestyle physical activity questionnaire [26]	Brunel PAQ	9	SA	Cs.V	2005	18 to 73	7
Community Healthy Activities Model Program for Seniors [10]	CHAMPS	41	SA	n.s.	2001	65 to 90	7
The European Prospective Investigation into Cancer Study-Norfolk [27]	EPIC-Norfolk	77	SA	Cr.V	2002	45 to 74	7 and 365
The European Prospective Investigation into Cancer Study-Short Form [28]	EPIC-s	11	SA	Cr.V	2003	20 to 70	7
Global Physical Activity Questionnaire [29]	GPAQ	16	SA	Cr.V	2003	18 to 75	7
International Physical Activity Questionnaire (Long Version) [30]	IPAQ-Long	27	SA, IB	Cr.V	1999	18 to 65	7
Minnesota Leisure Time Physical activity Questionnaire [31]	Minnesota LTPAQ	60	IB	Cr.V	n.s.	25 to 75	365
Morgenstern Physical Activity Questionnaire [32]	PAQ-M	14	SA	Cr.V	2011	n.s	7
Physical activity scale for the elderly [33]	PASE	19	SA	Cr.V	1993	65 and older	7
Rapid Assessment of Physical Activity [34]	RAPA	12	SA	Cr.V	2006	50 and older	7
Stanford Brief Activity Survey [35]	SBAS	10	n.s.	Cr.V	2006	60 to 69	1 to 7
Short Questionnaire to assess health-enhancing physical activity [36]	SQUASH	11	SA	Cr.V	2002	18 to 65	7
Yale Physical Activity Survey [9]	YPAS	36	SA	Cr.V	1988	60 to 86	7
Physical Activity Questionnaire from the Zutphen Cohort of the Seven Country Study [37]	Zutphen	28	n.s.	Cr.V	1997	65 to 84	7

# - number; SA – Self-administered; IB – Interview-based; o.v. – original version; Cr.V. – Criterion Validity; Ct.V. – Content Validity; Cs.V. – Construct Validity; n.s. – not specified.

### The International Classification of Functioning, Disability and Health (ICF)

The ICF is an international classification system developed by the World Health Organization [38]. The four ICF components (body functions, body structures, activity and participation, and environmental factors) consist of 1454 categories that are hierarchically arranged into chapters and levels. The biopsychosocial approach of the ICF provides a conceptual basis for the definition and measurement of functioning, disability and health. With its systematic coding scheme, the ICF functions as a common language to describe health and health-related states. All categories are defined by a unique code made up of (1) a letter representing the ICF component (2) followed by a numeric code representing the chapter level (first digit), the second level (next two digits) and the third level (fourth digit) (see Table 2).

**Table 2:** **Example of the hierarchical taxonomy system of the ICF**

**Code**	**Category**	**Level**
d	Activities and Participation	Component level
d4	Mobility	1^st^ level item
d450	Walking	2^nd^ level item
d4501	Walking long distances	3^rd^ level item

The following ICF codes were choosen for the linking procedure in equivalence to the four domains of the conceptual framework of Pettee-Gabriel et al. [16] to ensure common ground:

- Occupational activities are linked to d850
- Domestic activities are linked to ICF codes equal to d640 and d650 or higher
- Transport activities are linked to ICF codes equal to d470 and d450 or higher and
- Leisure-time activities are represented mainly through ICF codes equal to d920 or higher

Existing rules have been used to link content from instruments to the described hierarchical coding system [39,40] so that PAQ could be compared on a content basis.

### Linking procedure

Each item (n = 414) contained in the PAQ was linked to a code in the ICF. The linking procedure was conducted separately by two health professionals who were familiar with the ICF using established linking rules [39,40]. A third health professional was consulted to define the best fitting ICF code when the two primary health professionals did not agree on an item.

The main goal of this study was to analyze and discuss the PAQ content. For comprehensibility reasons ICF codes from the component *Activities and Participation* (d) in the first part (*Functioning and Disability*) of the ICF have been used. All concepts of the second part (*Contextual Factors*) were excluded to assure a solely activity-based analysis.

Three specific linking rules have to be noted because they were frequently present during the linking process. If items contained multiple concepts e.g., cited examples, then each concept was linked to the ICF. Therefore, one item can be linked to more than one ICF category. For example, item number 9 of the *EPIC-s* [28] “Housework such as cleaning, washing, cooking, child care etc.” was linked to d640 (“housework”), d6402 (“cleaning”), d6400 (“washing”), d630 (“cooking”) and d660 (“child care”). In contrast, if an item was not assignable because of insufficient information or because it was too general to allow an assignment, the item was classified “nd” (not definable). For example, item number 1 of the *RAPA* [34] “I rarely or never do any physical activities” was classified as “nd”. Items that were not covered by the ICF were classified as “nc” (not covered) such as item number 26 of the *Zutphen* [37] “What do you think of your pace compared with men of your age?”.

### Reliability analysis

For each instrument agreement between the two health professionals was calculated with MedCalc (Version 12.7.5, MedCalc Software bvba, Ostend, Belgium) for the second and third ICF levels using kappa statistics [41]. For these purposes the number of agreements and non-agreements were counted. Based on Altman [42], a kappa between 0.81 to 1. indicated very good, between 0.61 to 0.8 good, between 0.41 to 0.6 moderate, between 0.21 to 0.4 fair and below 0.2 poor agreement.

## Results

Overall, the two raters linked 414 items of 18 PAQ to 62 different ICF categories and 598 ICF codes. Additionally, 35 items were classified as “nd”, and 10 items were not covered by the ICF (“nc”). The kappa statistics as well as the corresponding confidence intervals showed moderate to very good agreement for 16 of the 18 PAQ, ranging from 0.488 to 1.00. The agreement for items of the *Brunel PAQ* [26] (kappa: 0.100) and the *RAPA* [34] (0.014) was poor (Table 3).

**Table 3:** **Estimated k coefficient and the bootstrapped confidence intervals at the 2** ^**nd**^ **and 3** ^**rd**^ **ICF levels of coding**

	**2nd level**		**3rd level**	
**Questionnaire**	**Kappa**	**CI (95%)**	**Kappa**	**CI (95%)**
7-day PAR	1.000	1.000 – 1.000	1.000	1.000 – 1.000
AAS	0.689	0.471 – 0.908	0.664	0.451 – 0.877
Baecke modified	0.651	0.410 – 0.891	0.603	0.383 – 0.823
BRFSS	0.488	0.124 – 0.853	0.488	0.124 – 0.853
Brunel PAQ	0.100	0.066 – 0.266	0.100	−0.066 – 0.266
CHAMPS	0.736	0.606 – 0.867	0.625	0.496 – 0.754
EPIC Norfolk	0.827	0.749 – 0.904	0.727	0.640 – 0.815
EPIC-s	0.667	0.468 – 0.866	0.454	0.257 – 0.650
GPAQ	0.950	0.855 – 1.000	0.861	0.717 – 1.000
IPAQ-Long	0.668	0.532 – 0.804	0.577	0.447 – 0.708
Minnesota LTPAQ	0.660	0.536 – 0.784	0.542	0.420 – 0.664
PAQ-M	0.874	0.760 – 0.988	0.783	0.665 – 0.901
PASE	0.777	0.631 – 0.923	0.596	0.450 – 0.741
RAPA	0.014	−0.017 – 0.045	0.043	−0.014 – 0.100
SBAS	0.836	0.717 – 0.955	0.649	0.507 – 0.791
SQUASH	0.830	0.678 – 0.982	0.580	0.386 – 0.774
YPAS	0.787	0.656 – 0.981	0.612	0.478 – 0.747
Zutphen	0.633	0.470 – 0.795	0.605	0.447 – 0.763

In total, 10 of 18 questionnaires contained ‘not definable’ items. The 35 items classified as ‘nd’ mainly originated from items asking about time spent being physically active at different intensity levels. Because the understanding of physical activity is very broad, a specific activity or context could not be identified. The majority of items (83.37%) of the *RAPA* [34] were considered ‘nd’. Other instruments showing a noticeable amount of ‘nd’ items were the *7-Day-PAR* [22] (50%) and the *Zutphen* [37] (21%).

Most linked ICF codes (98.8%) were related to the levels *mobility* (d4), *domestic life* (d6), *major life areas* (d8) and *community, social and civic life* (d9). 405 of 598 ICF codes could be found in the levels *mobility* (d4) and *community, social and civic life* (d9). Sport activities in general (d9201), *swimming* (d4554) and *running* (d4552) represented 43.72% of all linked ICF codes, followed by *walking* (d450) and *walking short* and *long distances* (d4500 and d4501) with 14.25%. Domestic activities, such as *doing laundry* (d6400), *doing kitchen work* (d6401), *cleaning the household* (d6402 and d6403) and *disposing garbage* (d6405), were less frequently linked (9.18%). Similar results were found for activities, such as *taking care of others* (d6600, d6601, d6604) with 2.89%, *taking the stairs* (d4453) with 3.14%, *using a bicycle* (d4750) with 7.00% and activities implying upper body movement, such as *lifting and carrying objects* (d430, d4300, d4301) with 5.7%.

In addition to analyzing the amount of identified ICF codes, it is important to look at the number of questionnaires containing those ICF codes because this value represents the degree of discrepancy between questionnaires. *Sports* (d9201) is the only ICF concept that is covered by each instrument. Depending on the ICF code and level, *walking activities* and *yard work* are investigated by most instruments (11 to 14 instruments), whereas domestic activities are asked about considerably less frequently (2 to 10 instruments). Occupational activities such as *paid work* (d850) and *unpaid work* (d855) are defined by 6 to 11 instruments and *care-taking activities* (d6600, d6601, d6604) are covered by 1 to 6 instruments (Table 4).

**Table 4:** **Frequency of items in each PAQ linked to an ICF category**

	**7-day PAR**	**AAS**	**Baecke modified**	**BRFSS**	**Brunel PAQ**	**CHAMPS**	**EPIC Norfolk**	**EPIC-s**	**GPAQ**	**IPAQ-Long**	**Minnesota LTPAQ**	**PAQ-M**	**PASE**	**RAPA**	**SBAS**	**SQUASH**	**YPAS**	**Zutphen**
**Total # of questionnaire items**	**10**	**13**	**12**	**8**	**9**	**41**	**77**	**11**	**16**	**27**	**60**	**14**	**19**	**12**	**10**	**11**	**36**	**28**
**Total # of ICF categories**	**14**	**20**	**15**	**11**	**9**	**52**	**98**	**23**	**23**	**43**	**72**	**44**	**37**	**2**	**44**	**17**	**48**	**26**
Learning & Applying Knowledge																		
d166: Reading						1							1		2			
d170: Writing															1			
Communication																		
d330: Speaking															1			
d3601: Using communication device						1												
Mobility																		
d4100: Lying down							1											
d4103: Sitting									1									
d4104: Standing							1											
d4150: Maintaining a lying position	2																	2
d4151: Maintaining a squatting position							1											
d4152: Maintaining a kneeling position							1											
d4153: Maintaining a sitting position							2	1	1	2		4	2		2	1	2	
d4154: Maintaining a standing position							4	1				1	1		1	1	1	
d430: Lifting and carrying objects							1					1	2		2			
d4300: Lifting							2			2						1		
d4301: Carrying in the hands							2	1	2	3	1				2	1	1	
d4402: Manipulating																	1	
d445: Hand and arm use												2	2		2			
d4450: Pulling						1					1							
d4451: Pushing							1											
d4453: Turning or twisting hands or arms								1										
d450: Walking				1		5	3				2	2	4		3		3	5
d4500: Walking short distances							2	1		1		1	1				1	
d4501: Walking long distances		3						1	2	6	3	2			1	1	4	1
d4502: Walking on different surfaces						1	1				1							
d4551: Climbing stairs		1	1				4	1		1	2		1				1	1
d4552: Running		2		1		1	2	1	1	1	2	1	1		3			
d4554: Swimming		2				2	2	1	1	2	2	1	1		1		1	
d460: Moving around in different locations																	1	
d4601: Moving around outside the home*													1					
d470: Moving around using transportation			2															
d4701: Using human-powered vehicles										2								
d4702: Using public motorized transportation							2			2								
d4750: Driving human-powered transportation		2				2	5	1	3	4	1		2		2	2	1	4
d4751: Driving motorized vehicle						1	4			2	1	1			3			
Domestic life																		
d6200: Acquisition of goods and services			2			1	2	1				1						
d6300: Preparing simple meals																		
d6301: Preparing complex meals			1				1	2								1	2	
d640: Doing housework	3										1	2			1			2
d6400: Washing and drying clothes*							1										1	
d6401: Cleaning cooking area and utensils			1				1					1				1	1	
d6402: Cleaning living area			3			2	1	2		2		1	2		1	1	2	
d6403: Using household appliances												1					1	
d6405: Disposing garbage			1														2	
d650: Caring for household objects	3																	1
d6500: Making and repairing clothes			1				1									1	2	
d6501: Maintaining dwelling and furnishings						1	1	1			4	2	1		1		2	1
d6503: Maintaining vehicles						1						1			1		1	
d6505: Taking care of plants, indoors and outdoors		2		1		2	4	1		4	5	3	2		4	1	4	2
d6506: Taking care of animals																		1
d6600: Assisting others with self-care							2	2				1	1			1	1	
d6601: Assisting others in movement						1						1					1	
d6604: Assisting others in nutrition			1															
Major life areas																		
d850: Remunerative employment	3				1		14	3	6	5	3	4	5		4	2		
d855: Non-remunerative employment						1	1			1		4	1			1		
Community, social and civic life																		
d910: Community life						1												
d920: Recreation and leisure											7							
d9200: Play						1						1			1			
d9201: Sports	3	8	2	8	8	18	25	1	6	3	36	3	5	2	5	1	10	5
d0902: Arts and culture						3	1											
d9203: Crafts						1						1	1				1	
d9204: Hobbies							1					1						1
d9205: Socializing						3	1											
d9300: Organized religion						1												
Not definable - physical activity	5		2			1	7		1				1	10		1	1	6
Not classified		5	1				3											

*Due to the length, the original ICF category is truncated. Full length and description can be found in the ICF [38].

The results of the linking procedure indicate that only 5 (*EPIC-Norfolk* [27], *EPIC-s* [28], *IPAQ-Long* [30], *PAQ-M* [32], *PASE* [33]) out of 18 questionnaires Table 4) contain items referring to all four domains. Taking the number of items into account, the *EPIC-Norfolk* [27] (n = 77 items) showed a high variability of ICF codes (n = 98) covering all four domains and simultaneously having a strong focus on low-intensity, functional physical activities such as household chores and care-taking activities. A high variability of ICF concepts (n = 44) but fewer items (n = 14) was found in the *PAQ-M* [32], which also covered all four domains. The remaining 13 instruments showed a large discrepancy in the domestic and occupational domains, whereas leisure-time activities, mainly due to the activity *sports* (d9201), are covered by each instrument.

Another important aspect of a person’s total physical activity behavior is their sedentary behavior. Sedentary activities, such as *sitting* or *lying*, increase with age and can have significant health-compromising effects independent of a person’s physical activity behavior [43]. The assessment of sedentary activities helps to appropriately appraise the health-enhancing effect of physical activity. Overall sedentary activities are clearly underrepresented with 5.55% of 598 ICF codes. 9 out of 18 questionnaires acquire information about *sitting* (d4103, d4153), and 2 out of 18 questionnaires include items referring to *lying* (d4100, d4150). 7 questionnaires (*AAS* [23], *Baecke modified* [24], *BRFSS* [25], *Brunel PAQ* [26], *CHAMPS* [10], *Minnesota LTPAQ* [31] and *RAPA* [34]) do not ask about any sedentary activities.

## Discussion

Each instrument included in this study was validated against different criteria. When considering validity, not only are criterion and construct validity important but also content validity because it has a major impact on the outcome variable [17]. To the authors’ knowledge, PAQ for the elderly have not been analyzed regarding their content on an item-based level. Despite methodological challenges, the ICF provides a comprehensive framework to compare and evaluate the content of PAQ. Overall, the results of the content analysis revealed inconsistencies in the understanding of physical activity between instruments and, subsequently, heterogeneity in its assessment.

Major differences were found on the domain level. Only 4 PAQ clearly covered the four recommended physical activity domains *leisure time, occupation, transport* and *household*. The majority of questionnaires contain domains that concentrate on the quality of an activity (e.g., “walking”), summarize certain activities under a broader domain (e.g., “doing odd jobs”, “do-it-yourself-activities” or “lifestyle activities”) or examine activities from an energy-based perspective (e.g., “light”, “moderate” or “vigorous” activities). This broad spectrum of physical activity domains reflects the high variability in the physical activity behavior in the elderly and underlines the necessity of assessing other relevant activities in addition to moderate and vigorous intensity activities [44]. At the same time, the differences indicate the need for a standardized conceptual framework of physical activity. Within the analyzed instruments, the occupational domain seemed to be used inconsistently, implying that this domain might be dispensable for a majority of people but at once relevant for self-employed, part-time or volunteer workers. For the sake of completeness and comparability, the adherence to minimum requirements, such as the assessment of the four standardized domains – *occupation*, *household*, *leisure time* and *transport* – is essential regardless of age [7].

In view of the ICF codes, the lack of agreement of the understanding of physical activity becomes even more evident as the differences on the domain level are aggrandized at the item level. Based on the linkage results, there are a few items, such as *sports* (d9201) or *walking activities* (d450, d4500, d4501), that assess the same content and are covered by most questionnaires. Overall, the majority of ICF codes vary in quantity and quality. In this case, quality stands for the ICF level on which the items were linked to. While some questionnaires ask for activities which were identified on the 2^nd^ level, others differentiate an activity more precisely on the 3^rd^ level. Additionally, the results indicate that the selected measures mainly focus on activities that are associated with higher energy expenditure. Light intensity activities that serve a more functional purpose, such as *household chores* (d640 and higher, d650 and higher) or *recreational activities*, such as *hobbies* (d9204) or *social activities* (d9205), are considered remarkably less often, even though light intensity activities represent the largest part of daily physical activity [6,45]. Healy et al. [45] report that over 90% of waking hours are “spent either in sedentary or in light-intensity activity” (p. 371). Although the health benefits of moderate to vigorous intensity activities cannot be replaced, the benefits of light-intensity activities are evident and can be observed in the reduction of sedentary time [5] and in increased total daily energy expenditure [45]. Additionally, living a physically active lifestyle preserves the physical functionality of the elderly [3] and therefore substantially contributes to a person’s quality of life [46].

There are different reasons for the lack of agreement regarding the definition of physical activity and its assessment via self-report methods. First, the context and the scientific era in which an instrument was developed have to be considered. Developing from a fitness paradigm that mainly emphasized vigorous intensity, performance-enhancing activities such as aerobic power or endurance and muscular strength activities, the health paradigm also focused on moderate intensity activities and accumulation of 30 minutes of them throughout the day on most days of the week [8]. With regard to the physical activity behavior of the elderly, both paradigms examine a small window of physical activity, resulting in floor effects. From these energy-related perspectives, single approaches considering the predominant sedentary and light intensity activities of older adults originated e.g., the *CHAMPS* [10], the *PAQ-M* [32] or the *YPAS* [9]. Although these derived questionnaires represent a broader and more accurate range of activities, they were developed for different purposes (e.g. some questionnaires concentrate solely on a certain domain like occupational or lifestyle activities, whereas others focus on specific intensity categories) and therefore did not adhere to a conceptual framework of physical activity, which may have caused the identified content-based differences.

Furthermore, the use of energy-based domains such as light, moderate or vigorous intensity physical activity, e.g., the *7-Day PAR* [22], is less conclusive and leaves room for interpretation of which actual activities to assess. This is relevant for researchers developing questionnaires as well as for test subjects with different understandings of the terms *light*, *moderate* and *vigorous intensity*.

### Methodological considerations

In addition to the discussion of the results, it is important to consider some methodological limitations of this investigation. Some concepts were interpreted differently by two raters and therefore linked to two different ICF codes. Although a final consensus was reached, linking decisions were made for each item alone without additional context information about the instrument. Items assessing *walking activities* with additional information about time (e.g., “for at least 10 minutes”) were perceived differently and therefore linked to either d450 or d4501. The same is true for the term “physical activity”, which in some questionnaires was used within a certain domain such as *recreation*, *leisure time*, *household* or *occupation* but was not clearly phrased within the item.

Moreover, questionnaires and their items were listed consecutively for the linking process leading to the assumption (one rater) that a question’s content could be connected to the previous question and therefore impacting the amount of identified concepts. *IPAQ-Long* [30] questions number 18 (“[…] you do moderate activities like carrying light loads, washing windows, scrubbing floors and sweeping inside your home?”) and 19 (“[…] on one of those days doing moderate physical activities inside your home?”) exemplify this fact.

A general problem during the linkage and the discussion of those results involved questionnaires containing energy-related items such as “light”, “moderate” or “vigorous” intensity activities. Those questionnaires caused an inevitable and predictable discrepancy compared to questionnaires asking about specific activities.

## Conclusions

The main goals of this article were to analyze the content of PAQ for the elderly, discuss the findings with regard to a conceptual framework and derive recommendations for practitioners and researchers. The results indicated a lack of agreement in terms of assessing levels of physical activity by the elderly with self-report measures. This fact becomes evident when looking at the quantity and variability of physical activity domains and actual activities. There are multiple possible causes for the discrepancies between instruments such as the context, the time and the intended purpose of the questionnaires.

At the same time, there is a lack of consistency about the use of light intensity physical activities and their relation to health-promoting effects in the elderly. Up to now, the analyzed questionnaires derived from a somatic-based perspective, where activities with higher energy expenditure rate are in the foreground. In contrast, the content of various instruments such as the *CHAMPS* [10] or the *YPAS* [9] indicate more biopsychosocial approaches where light intensity, functional activities in particular are considered. Despite the controversial discussion of the assessment of light intensity physical activities with self-report measures [5,42], light intensity physical activities represent one of the largest parts of daily physical activity in the elderly and therefore reflect a more accurate picture of an older person’s physical activity behavior.

This being said, future research should add a biopsychosocial perspective to the existing energy-based approaches. Within the conceptual framework of Pettee Gabriel et al. [16], the energy expenditure and components of physical fitness are declared as the main outcome parameters of physical activity. Due to the mentioned evidence of physical functioning, the health-promoting effects of light intensity physical activity such as a person’s health-related quality of life should be considered within the framework. To draw causal conclusions, compare the results to different populations and derive general recommendations, the assessment of physical activity, especially functional, light intensity physical activity, should be standardized in terms of domains and actual activities. At the same time, questionnaires should consider a broader range of activities for the elderly because age-related degradation processes tend to develop in an asynchronous manner. Therefore, it is possible that one person may still be working part-time or voluntarily and participates in vigorous sports activities while another person at the same age is only able to pursue light to moderate intensity activities.

The results indicated an insufficient assessment of sedentary behavior. Future research, in particular when developing PAQ, should focus on the implementation of questions regarding sedentary behavior as proposed in the conceptual framework by Pettee Gabriel et al. [16]. This is partly important because health-promoting effects can be negated by the health-compromising effects of sedentariness [17,43]. The accurate determination and interpretation of the effects of physical activity can only be achieved by assessing both physical activity and sedentary behavior.

The assessment of physical activity is one of the most important aspects in the context of health promotion. Not only are interventions evaluated but also large populations are monitored and state-wide health policies and health care systems are aligned based on the assessed results. PAQ are cost-effective and easily applicable in a large population but, depending on the content, can be at the same time misleading. Many of the PAQ included in this study do not claim to cover all aspects of physical activity and functioning, e.g. the 7-day PAR [22] is intended to assess leisure and occupational physical activity only. In general, each PAQ aims to cover a particular aspect of physical activity. Therefore, instruments assessing physical activity levels should be selected carefully and the results interpreted appropriately with regard to context, population and purpose.
